# Supplementary material for: In Vivo Mechanical Assessment of Cortical Bone Rigidity Enhances Fracture Discrimination Beyond DXA in Postmenopausal Women
Source: medRxiv. 2025 Aug 29:2025.08.28.25334655. Preprint. [Version 1] doi: 10.1101/2025.08.28.25334655 (PMC12407662; doi:10.1101/2025.08.28.25334655)
Supplement: Supplementary file 1 [file NIHPP2025.08.28.25334655v1-supplement-1.pdf]

Supplemental Table 1. Inclusion and exclusion criteria for study participants in both target population groups (i.e., fragility fracture cases and controls).

Inclusion Criteria Common Across Both Cases & Controls	Inclusion Criteria Specific to Fragility Fracture Cases	Inclusion Criteria Specific to Controls
Females	Had experienced a fragility fracture of the arms (including wrist/carpal fractures) or legs (including hip, pelvis, and ankle fractures) after the age of 50 years. Insufficiency and stress fractures, as well as fractures of the spine, digits, toes, or face, were not included. A fragility fracture was operationally defined based on self-report of an arm or leg fracture caused by falls from a height <6 inches. A fragility fracture was not counted if it was associated with 1) running, bicycling or other similar fast-moving activity such as sports, 2) being struck by a falling or otherwise quickly moving heavy object, or 3) a motor vehicle accident.	Self-reported no history of fragility fractures at any time, or traumatic fractures at any site after age 40, excluding fractures of the digits, toes, or face.
50-80 years of age		
Self-reported that their last menses occurred at least 24-months prior to enrollment.		
BMI between 18.5 and 35 kg/m^2.		
In the opinion of the site principal investigator the study participant is physically able to safely participate in the study activities		Does not self-report losing more than 3.8 cm (1.5 inches) in stature in the prior 15 years.
Able to provide informed consent		

Exclusion Criteria Common Across Both Cases & Controls
Use of systemic glucocorticoids for more than 6-months in the prior one year
Self-reported diseases that could interfere with bone metabolism. For example, osteomalacia, bone cancer, myeloma, Paget's disease, hyper parathyroidism, hyperthyroidism not treated, severe renal (e.g., stage 4+ chronic kidney disease, history of dialysis, kidney transplant, etc.), hepatic insufficiency, or prolonged immobilization (more than 2 months in the previous year)
Self-reported type 1 diabetes
Self-reported being told by a physician that they have a terminal illness
Had had bilateral hip replacements
The subject was excluded if they answered yes to the following question: Do you have an active rotator cuff tear, had shoulder surgery in the past 12-months, or experience severe shoulder, wrist, or elbow joint pain on a regular basis?
Persons living in a nursing home (those living in assisted living or independent housing were not excluded)
Unable to communicate because of severe hearing loss or speech disorder
If, in the opinion of a site principal investigator, a study participant was inappropriate for the scientific purposes of this study. For instance, a high fall risk patient due to an existing neurological disorder (e.g., Parkinson's disease, ALS, etc.) would be excluded
Failure to provide informed consent

medRxiv preprint doi: https://doi.org/10.1101/2025.08.28.25334655; this version posted August 29, 2025. The copyright holder has placed this preprint (which was not certified by peer review) in the Public Domain. It is no longer restricted by copyright. Anyone can legally share, reuse, remix, or adapt this material for any purpose without crediting the original authors.

Supplemental Table 2. Fracture Characteristics Among Case Participants (mean time since fracture: 5.0 ± 4.2 years).

Fracture Location	Fracture Site	n (% of participants)
**Upper Extremity**		
	Radius	31 (28%)
	Humerus	19 (17%)
	Ulna	12 (11%)
	Carpal	11 (10%)
	Clavicle	1 (1%)
**Lower Extremity**		
	Fibula	22 (20%)
	Tibia	12 (11%)
	Hip (proximal femur)	9 (8%)
	Pelvis	2 (2%)
	Femur (distal)	1 (1%)
**Total**		

Percentages represent the proportion of case participants (n = 109) who experienced a fracture at each site. Because some individuals sustained more than one fracture, the total number of fractures exceeds the total number of cases and percentages sum to >100%.

medRxiv preprint doi: https://doi.org/10.1101/2025.08.28.25334655; this version posted August 29, 2025. The copyright holder has placed this preprint (which was not certified by peer review) in the Public Domain. It is no longer restricted by copyright. Anyone can legally share, reuse, remix, or adapt this material for any purpose without crediting the original authors.

Supplemental Table 3. Osteoporosis pharmacotherapy exposure among study participants.

Drug Class	n (%)
**Bisphosphonates**	
Bisphosphonates — 1st Generation	2 (1.4%)
Bisphosphonates — 2nd Generation	76 (52.8%)
Bisphosphonates — 3rd Generation	36 (25.0%)
**Monoclinal Antibodies**	
Denosumab	13 (9.0%)
Romosozumab	5 (3.5%)
**PTH Analogs**	10 (6.9%)
**SERM**	1 (0.7%)

Percentages are expressed as proportion of total pharmacotherapy exposures.

medRxiv preprint doi: https://doi.org/10.1101/2025.08.28.25334655; this version posted August 29, 2025. The copyright holder has placed this preprint (which was not certified by peer review) in the Public Domain. It is no longer restricted by copyright. Anyone can legally share, reuse, remix, or adapt this material for any purpose without crediting the original authors.

Supplementary Table 4. Participant Characteristics by Fracture Status in the Osteoporosis Treatment-Naïve Subset.

Variable	Fracture Cases (n = 62) Mean (SD)	Controls (n = 197) Mean (SD)	Cohen's d Effect Size (CI)	P Value
**Demographics/Clinical**				
Age, years	65.7 (5.8)	65.8 (6.5)	0.01 (-0.28, 0.29)	0.96
Height, cm	164.1 (6.0)	162.7 (6.3)	0.22 (-0.07, 0.51)	0.13
Weight, kg	72.3 (12.8)	70.2 (11.7)	0.18 (-0.07, 0.50)	0.23
Body Mass Index, kg/m²	26.7 (4.1)	26.4 (3.9)	0.08 (-0.20, 0.37)	0.58
Charlson 10-Year Survival, %	86.32 (13.4)	85.4 (11.5)	0.08 (-0.21, 0.37)	0.58
No Bone Rx History, n (%)	62 (100)	197 (100)	.	.
**Physical Function**				
Hand Grip Strength, kg	23.0 (6.3)	24.6 (6.3)	0.25 (-0.04, 0.54)	0.09
Usual Gait Speed, m/sec	1.07 (0.20)	1.11 (0.19)	0.21 (-0.08, 0.50)	0.16
Timed Up and Go, secs	9.1 (1.9)	8.9 (2.0)	0.12 (-0.17, 0.40)	0.43
Four Square Step Test, secs	8.9 (2.3)	8.4 (1.8)	0.23 (-0.06, 0.52)	0.12
**CBMT Flexural Rigidity**				
Ulna EI, N·m²	**20.7 (7.7)**	**25.1 (8.9)**	0.52 (0.23, 0.81)	**<0.001**
Ulna EI Relative to Weight, N·m²/kg	**0.29 (0.09)**	**0.36 (0.11)**	0.68 (0.38, 0.97)	**<0.001**
**DXA Areal BMD**				
Lumbar Spine, g/cm²	**0.93 (0.15)**	**0.98 (0.16)**	**0.30 (0.01, 0.59)**	**0.04***
Total Hip, g/cm²	0.80 (0.12)	0.84 (0.13)	0.25 (-0.04, 0.54)	0.09
Femoral Neck, g/cm²	0.69 (0.09)	0.72 (0.12)	0.21 (-0.08, 0.50)	0.16
1/3rd Radius, g/cm²	0.63 (0.09)	0.63 (0.09)	0.00 (-0.29, 0.29)	0.99

Continuous variables are presented as mean (SD). Group comparisons between fracture cases and controls were performed using independent-samples t-tests. CBMT indicates Cortical Bone Mechanics Technology; EI, flexural rigidity; BMD, bone mineral density; DXA, dual-energy x-ray absorptiometry; Rx: Prescription; Hx, history. Effect sizes are presented as Cohen d for continuous variables. The No Prior Bone Medication Use variable was not compared, as all subgroup participants had no prior use. CI, confidence interval. All data from the non-dominant limb.

medRxiv preprint doi: https://doi.org/10.1101/2025.08.28.25334655; this version posted August 29, 2025. The copyright holder has placed this preprint (which was not certified by peer review) in the Public Domain. It is no longer restricted by copyright. Anyone can legally share, reuse, remix, or adapt this material for any purpose without crediting the original authors.

Supplementary Table 5. Participant Characteristics by Fracture Status in the Subset with Non-Osteoporotic BMD.

Variable	Fracture Cases (n = 92) Mean (SD)	Controls (n = 223) Mean (SD)	Cohen's d Effect Size (CI)	P Value
**Demographics/Clinical**				
Age, years	67.1 (6.1)	66.5 (6.4)	0.10 (-0.14, 0.34)	0.69
Height, cm	163.5 (6.2)	162.6 (6.4)	0.14 (-0.11, 0.38)	0.90
Weight, kg	69.7 (12.6)	68.9 (11.6)	0.07 (-0.17, 0.31)	0.82
Body Mass Index, kg/m²	26.0 (4.2)	26.0 (3.8)	0.01 (-0.17, 0.31)	0.52
Charlson 10-Year Survival, %	84.4 (14.2)	84.4 (11.7)	0.00 (-0.25, 0.26)	0.98
No Bone Rx History, n (%)	**50 (58.8)**	**162 (77.5)**	**0.19**	**<0.001**
**Physical Function**				
Hand Grip Strength, kg	22.1 (6.5)	23.7 (6.3)	0.26 (0.01, 0.50)	0.72
Usual Gait Speed, m/sec	**1.08 (0.22)**	**1.11 (0.19)**	**0.14 (-0.11, 0.38)**	**0.02**
Timed Up and Go, secs	9.1 (2.0)	8.9 (2.0)	0.12 (-0.13, 0.36)	0.41
Four Square Step Test, secs	**8.9 (2.3)**	**8.4 (1.8)**	**0.29 (0.05, 0.54)**	**0.003**
**CBMT Flexural Rigidity**				
Ulna EI, N·m²	**19.8 (7.1)**	**25.2 (8.8)**	0.64 (0.39, 0.90)	**<0.001**
Ulna EI Relative to Weight, N·m²/kg	**0.29 (0.09)**	**0.36 (0.11)**	0.74 (0.48, 0.99)	**<0.001**
**DXA Areal BMD**				
Lumbar Spine, g/cm²	0.97 (0.13)	1.00 (0.14)	0.22 (-0.03, 0.47)	0.25
Total Hip, g/cm²	0.82 (0.11)	0.84 (0.12)	0.16 (-0.09, 0.41)	0.31
Femoral Neck, g/cm²	**0.70 (0.09)**	**0.72 (0.11)**	**0.17 (-0.08, 0.41)**	**0.03**
1/3rd Radius, g/cm²	0.64 (0.08)	0.64 (0.09)	0.05 (-0.20, 0.29)	0.51

Continuous variables are presented as mean (SD). Group comparisons between fracture cases and controls were performed using independent-samples t-tests. CBMT indicates Cortical Bone Mechanics Technology; EI, flexural rigidity; BMD, bone mineral density; DXA, dual-energy x-ray absorptiometry; Rx: Prescription; Hx, history. Effect sizes are presented as Cohen d for continuous variables and as the Phi coefficient for the No Prior Bone Medication Use variable. CI, confidence interval. All data from the non-dominant limb.

medRxiv preprint doi: https://doi.org/10.1101/2025.08.28.25334655; this version posted August 29, 2025. The copyright holder has placed this preprint (which was not certified by peer review) in the Public Domain. It is no longer restricted by copyright. Anyone can legally share, reuse, remix, or adapt this material for any purpose without crediting the original authors.

Supplemental Table 6. Univariable Logistic Regression and AUC Results for Age and BMI in Discriminating Fracture Status in the Full Sample. Threshold-based metrics (cutoff, sensitivity, specificity, PPV, NPV) are omitted for predictors with non-significant discriminatory performance (AUC P > .05).

	Logistic Regression Results			ROC Curve Results							
**Predictor**	**OR Per SD**	**95% CI**	**P-Value**	**AUC**	**95% CI**	**P-Value**	**Cutoff**	**Sensitivity (%)**	**Specificity (%)**	**PPV (%)**	**NPV (%)**
**Age**	1.09	0.85, 1.42	0.51	0.53	0.42, 0.65	0.58	–	–	–	–	–
**BMI**	0.93	0.93, 0.94	0.62	0.48	0.35, 0.61	0.67	–	–	–	–	–

Odds ratios (ORs) are expressed per 1–standard deviation (SD) decrease in the predictor. ROC = Receiver operating characteristic curve. AUC = area under the ROC curve.

medRxiv preprint doi: https://doi.org/10.1101/2025.08.28.25334655; this version posted August 29, 2025. The copyright holder has placed this preprint (which was not certified by peer review) in the Public Domain. It is no longer restricted by copyright. Anyone can legally share, reuse, remix, or adapt this material for any purpose without crediting the original authors.

Supplemental Table 7. Multivariable Logistic Regression Models Predicting Fracture Status Using CBMT-Derived Flexural Rigidity Normalized to Body Weight with Adjustment for Age, BMI, and DXA-Derived BMD in the Full Sample.

		Logistic Regression Results			ROC Curve Results		
**Model**	**Predictors**	**OR Per SD**	**95% CI**	**P-Value**	**AUC**	**95% CI**	**P-Value**
**Relative CBMT EI Only (Univariable)**					**0.80**	**0.70–0.89**	**< 0.001**
	Relative CBMT EI	**0.57**	**0.42, 0.80**	**< 0.001**			
**Relative CBMT EI + Age and BMI**					**0.77**	**0.67-0.87**	**<0.001**
	Relative CBMT EI	**0.56**	**0.39, 0.81**	**<0.001**			
	Age	1.06	0.79, 1.42	0.69			
	BMI	0.81	0.60, 1.10	0.17			
**Relative CBMT EI + Lumbar Areal BMD**					**0.75**	**0.65, 0.85**	**< 0.001**
	Relative CBMT EI	**0.56**	**0.38, 0.82**	**< 0.01**			
	Lumbar Areal BMD	0.81	0.60, 1.09	0.15			
**Relative CBMT EI + Total Hip Areal BMD**					**0.75**	**0.65, 0.86**	**< 0.001**
	Relative CBMT EI	**0.60**	**0.42, 0.86**	**< 0.01**			
	Total Hip Areal BMD	0.81	0.59, 1.12	0.07			
**Relative CBMT EI + Femoral Neck Areal BMD**					**0.78**	**0.67, 0.88**	**< 0.001**
	Absolute CBMT EI	**0.60**	**0.42, 0.86**	**< 0.01**			
	Femoral Neck Areal BMD	0.87	0.65, 1.17	0.36			
**Relative CBMT EI + 1/3rd Radius Areal BMD**					**0.78**	**0.68, 0.88**	**< 0.001**
	Absolute CBMT EI	**0.58**	**0.41, 0.83**	**< 0.01**			
	1/3rd Radius Areal BMD	1.00	0.72, 1.37	0.48			

Odds ratios (ORs) are expressed per 1–standard deviation (SD) decrease in the predictor. ROC = Receiver operating characteristic curve. AUC = area under the ROC curve. CBMT = Cortical Bone Mechanics Technology; EI = Flexural rigidity; BMD = bone mineral density. All data from the non-dominant limb.

medRxiv preprint doi: https://doi.org/10.1101/2025.08.28.25334655; this version posted August 29, 2025. The copyright holder has placed this preprint (which was not certified by peer review) in the Public Domain. It is no longer restricted by copyright. Anyone can legally share, reuse, remix, or adapt this material for any purpose without crediting the original authors.
